# Unraveling the Genetic Link Between Endocrine Hormones and Psychiatric Disorders: An Atlas of Genetic Correlations

**DOI:** 10.3390/endocrines6030032

**Published:** 2025-07-02

**Authors:** James L. Li

**Affiliations:** 1 Department of Public Health Sciences, University of Chicago, Chicago, IL 60637, USA;; 2 Interdisciplinary Scientist Training Program, University of Chicago, Chicago, IL 60637, USA

**Keywords:** thyroid, sex hormones, psychiatric disorders, genetic architecture, genetic correlations

## Abstract

**Background/Objectives:**

Endocrine hormones play critical roles in regulating physiological processes, and previous studies have reported their associations with psychiatric disorders. Levels of endocrine hormones and the risk of developing psychiatric disorders are influenced by both genetic and non-genetic factors. However, the shared genetic basis underlying these associations remains largely unexplored. This study aims to dually evaluate the genetic correlations among endocrine hormones, including thyroid and sex hormones, as well as between endocrine hormone metrics and psychiatric disorders to identify potential shared genetic architectures.

**Methods:**

We obtained genome-wide association study summary statistics for six thyroid hormone metrics, three sex hormone metrics, and ten psychiatric disorders from predominantly European-ancestry populations. Genetic correlations were computed using linkage disequilibrium score regression after harmonizing variant data to ensure consistency across studies.

**Results:**

Significant genetic correlations were observed among thyroid and sex hormone metrics, indicating a strong shared genetic basis. Sex hormones exhibited multiple genetic correlations with psychiatric disorders, including negative correlations between sex hormone-binding globulin and attention-deficit hyperactivity disorder (ADHD) (*p* = 3.95 × 10^−12^) and major depressive disorder (*p* = 4.67 × 10^−5^), and positive genetic correlations with anorexia nervosa (*p* = 2.86 × 10^−12^) and schizophrenia (*p* = 2.00 × 10^−4^). Testosterone and estradiol had negative genetic correlations with ADHD and major depressive disorder, while testosterone had positive genetic correlations with anorexia nervosa and schizophrenia. Although thyroid hormone metrics did not exhibit Bonferroni-significant genetic correlations, nominal associations were observed, such as a negative genetic correlation between thyroid-stimulating hormone and major depressive disorder (*p* = 2.33 × 10^−2^).

**Conclusions:**

These findings suggest a shared genetic basis between endocrine hormones and psychiatric disorders, particularly for sex hormones. Future studies leveraging larger, more diverse populations are warranted to validate and extend the genetic correlations observed in this study.

## 1. Introduction

Endocrine hormones are critical for regulating various physiological processes in the human body by influencing pathways related to metabolism, growth and development, reproduction, and the body’s response to stress [1,2]. Previous epidemiological studies have established associations between levels of endocrine hormones and psychiatric disorders [2]. Among these associations, the co-occurrence of thyroid hormones and mental health disorders has been extensively reported, with several clinical observations of hypothyroidism including depression and cognitive impairment and even severe cases of thyroid failure linked to psychosis and schizophrenia [2,3]; in contrast, hyperthyroidism has been associated with a markedly high occurrence of psychiatric conditions including depression affecting 30–70% of cases and anxiety disorders affecting 60% of cases [3]. Additionally, levels of sex hormones have been linked with mental health disorders including androgen deficiency, which has been associated with depressive symptoms, impaired mental concentration, and memory loss [4], and estrogen deprivation, which has been associated with depression, sleep disruption, and anxiety [5,6].

Despite well-documented associations between endocrine hormones and psychiatric disorders in clinical and epidemiological studies, the underlying mechanisms remain unclear, as both genetic and non-genetic factors are known to influence hormone regulation [7] and psychiatric disease risk [8]. Genetic correlation quantifies the shared genetic basis between two traits by analyzing genome-wide association study (GWAS) summary statistics of common variants in the population. Although genetic correlations do not directly capture physiological mechanisms or causal relationships between traits, they have been used to identify the shared genetic architecture underlying complex traits and diseases, which may in part explain co-occurring conditions and associations observed in epidemiological studies [9,10]. However, thus far, the shared genetic basis between endocrine hormones and psychiatric disorders has been largely unexplored. Aiming to address this gap, we computed genetic correlations (1) among endocrine hormone metrics (including six thyroid hormone metrics and three sex hormone metrics), and (2) between endocrine hormones metrics and ten psychiatric disorders. To the best of our knowledge, this is the first study to systematically evaluate the shared genetic architecture between endocrine hormones and psychiatric disorders. By leveraging common genetic variants in the population, our analysis extends previous epidemiological findings by providing insight into the genetic basis underlying these associations.

## 2. Materials and Methods

### 2.1. Selection of Endocrine Hormones and Psychiatric Disorders for Analysis

Endocrine hormones that were included in this study have either hypothesized or established relationships with psychiatric disorders that have been identified in previous epidemiological and clinical studies. For instance, the hypothalamic–pituitary–gonadal (HPG) and hypothalamic–pituitary–thyroid (HPT) axes play central roles in neurodevelopment, cognitive function, and the regulation of mood [11–13]. As such, we analyzed hormones that are reported to be major regulators of the HPG axis, including sex hormone-binding globulin (SHBG), estradiol, and testosterone. Additionally, thyroid hormones including thyroid-stimulating hormone (TSH), free triiodothyronine (FT3), total triiodothyronine (TT3), and free thyroxine (FT4) have been reported as key players of the HPT axis, and we accordingly included them. We further included FT3/FT4 and TT3/FT4 ratios to capture the conversion efficiency of thyroid hormones—which may be physiologically relevant and not captured by single hormone metrics.

We selected psychiatric disorders to encompass a broad range of neurodevelopmental, anxiety, and mood disorders. These disorders include common conditions such as major depressive disorder, schizophrenia, ADHD, bipolar disorder, Type 1 bipolar disorder, and Type 2 bipolar disorder, as well as less common but still highly heritable conditions such as Tourette’s syndrome, obsessive–compulsive symptoms, anorexia nervosa, and panic disorder. Overall, all the endocrine hormones and psychiatric disorders included in this study were selected based on prior work indicating the presence of a substantial genetic basis, with many of these traits having previously reported genome-wide significant loci [14–23].

### 2.2. Obtaining GWAS Summary Statistics for Endocrine Hormones and Psychiatric Disorders

For all of the aforementioned endocrine hormones and psychiatric disorders, we obtained GWAS summary statistics involving individuals of predominantly European ancestry, and these studies are listed in Supplementary Table S1 [14–23]. GWAS sample sizes for endocrine hormone metrics ranged from around 15,000 to over 380,000 individuals, with TSH being the most well-powered thyroid hormone metric. GWAS sample sizes for psychiatric disorders ranged from around 10,000 to over 800,000 individuals, with disorders such as schizophrenia, ADHD, major depressive disorder, and bipolar disorder each involving over >200,000 individuals, with other disorders such as Tourette’s syndrome, anorexia nervosa, panic disorder, and obsessive–compulsive symptoms each involving <100,000 individuals. All the participating studies involved in the generation of these GWAS summary statistics previously obtained informed consent from participants and approval from their appropriate institutional review boards.

### 2.3. Harmonizing GWAS Summary Statistics

The genomic coordinates of all variants were converted to the GRCh37 human reference build using the liftOver software package [24]. Baseline and effect alleles for variants were standardized across studies, as well as their effect sizes. For variants with missing minor allele frequency annotations, we utilized allele frequencies from European individuals in the 1000 Genomes Project [25]. After this variant harmonization, we annotated variants with their rsID using the dbSNP database [26]. In order to maintain high consistency in the imputation quality of variants across all included analyzed phenotypes, we restricted variants to those included in the HapMap3 panel [27], which constitutes a set of common variants that tend to be well-imputed across studies.

### 2.4. Evaluating the Statistical Power for Calculating Genetic Correlations with Observed GWAS Sample Sizes

We utilized the GCTA-GREML [28] power calculator to compute the power for identifying genetic correlations between (1) endocrine hormone metrics and (2) endocrine hormone metrics and psychiatric disorders in this study utilizing the observed GWAS sample sizes for each trait. To calculate power with the GCTA-GREML power calculator, we utilized assumptions that the average heritability of each trait was 10%, pairwise genetic correlation was 15%, pairwise phenotypic correlation was 15%, the variance of the SNP-derived genetic relationship was 2 × 10^−5^, and we set the alpha significance threshold to 0.05.

### 2.5. Computing Genetic Correlations Between Endocrine Hormone Metrics and Psychiatric Disorders

We first used the LD score regression (LDSC) package [29] to estimate pairwise genetic correlations between different endocrine hormone metrics, and subsequently, between each hormone metric and each psychiatric disorder. To perform this analysis, we input the harmonized GWAS summary statistics along with linkage disequilibrium reference panels that had previously been generated using HapMap3 variants [27,29] from European individuals in the 1000 Genomes Project [25]. Since the GWAS summary statistics for the included endocrine hormone metrics and psychiatric disorders were developed using outcomes measured on varying scales (i.e., binary vs. continuous vs. transformed outcomes), the following results in the main text report only *p*-values and the directionality of each genetic correlation, rather than the magnitudes of each genetic correlation. We accounted for multiple testing by utilizing a Bonferroni threshold of significance to identify statistically significant genetic correlations (*p* < 0.05/number of unique tests), while also reporting nominal associations at *p* < 0.05 as being suggestive.

## 3. Results

### 3.1. Power Analysis for Detecting Genetic Correlations Using Observed GWAS Sample Sizes

Prior to examining the shared genetic architecture between endocrine hormones and psychiatric disorders, we performed power calculations based upon the sample sizes utilized in the GWAS of endocrine hormone metrics and psychiatric disorders included in this study. With these observed GWAS sample sizes, we identified that 24 of the 36 unique pairwise comparisons between endocrine hormone metrics had a power above 0.80 at an alpha significance threshold of 0.05; in particular, the 12 pairs with a power below 0.80 involved TT3, FT3/FT4, and TT3/FT4 hormone metrics that had relatively lower sample sizes ranging from 15,510 to 51,095 individuals. Furthermore, among the 90 unique pairwise comparisons between endocrine hormone metrics and psychiatric disorders, 64 pairs had a power above 0.80. Psychiatric disorders with limited sample sizes among the pairs with a power below 0.80, included panic disorder, Tourette’s syndrome, and obsessive–compulsive symptoms ranging from 10,240 to 33,943 individuals.

### 3.2. The Shared Genetic Architecture of Endocrine Hormones

**Figure 1.**
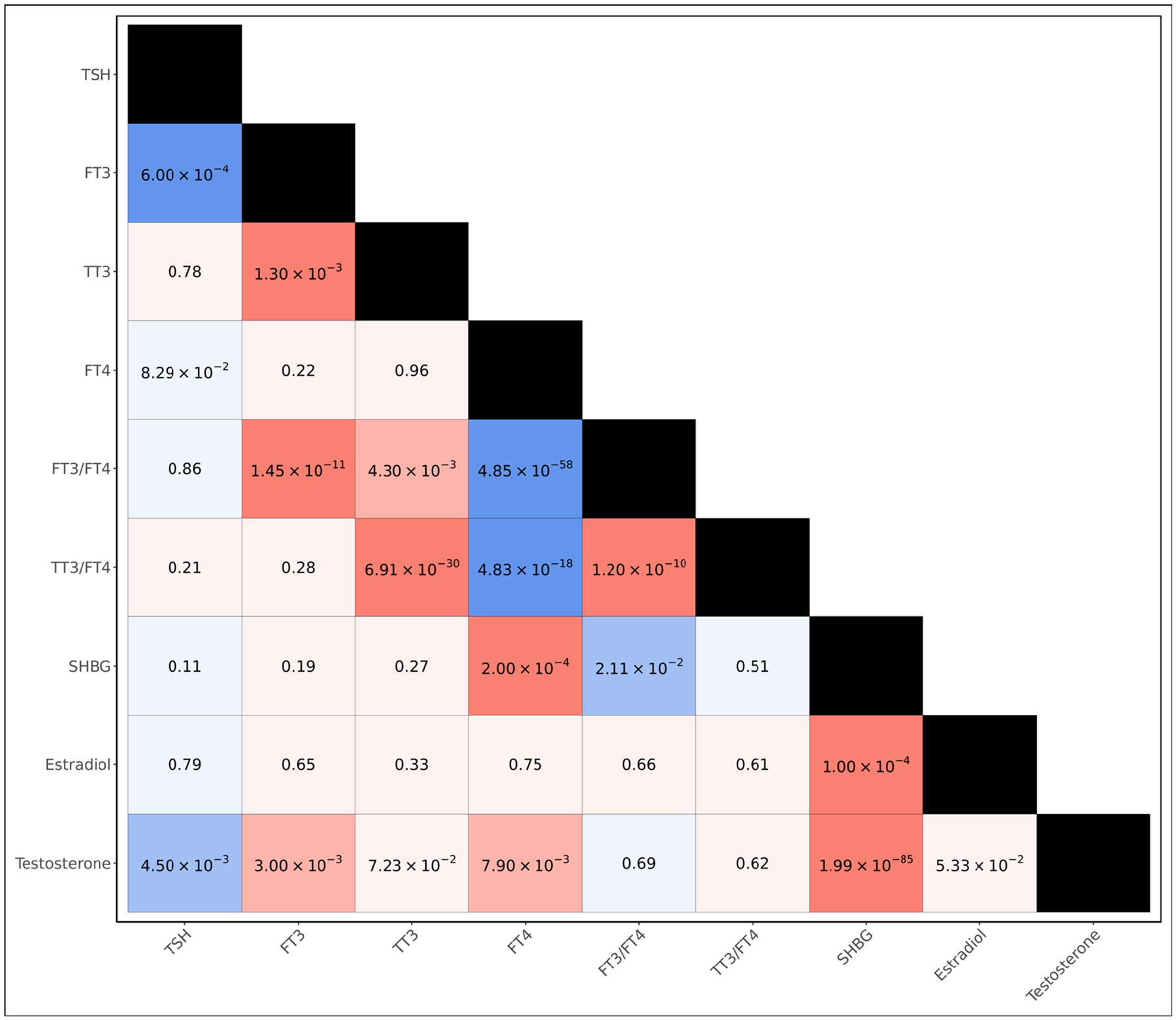
*p*-values of genetic correlations among different endocrine hormone metrics. Positive genetic correlations are colored in red and negative genetic correlations are colored in blue. Faint shading indicates a genetic correlation was not statistically significant (*p* ≥ 0.05). Moderate shading indicates a suggestive, nominally significant genetic correlation (*p* < 0.05). Dark shading indicates a genetic correlation remained significant after adjusting for multiple testing (Bonferroni-adjusted *p* < 0.05 or unadjusted-*p* < 1.39 × 10^−3^).

We first examined the shared genetic architecture of endocrine hormones by computing genetic correlations between each of the thyroid and sex hormone metrics (Figure 1, Supplementary Table S2). We observed that the genetic bases of thyroid hormone metrics tended to be strongly shared, with all six thyroid hormone metrics yielding statistically significant genetic correlations with at least one other thyroid hormone metric at the Bonferroni threshold of significance (*p* < 1.39 × 10^−3^); in particular, we observed negative genetic correlations between FT4 and both FT3/FT4 (*p* = 4.85 × 10^−58^) and TT3/FT4 (*p* = 4.83 × 10^−18^), and a positive genetic correlation between TSH and FT3 (*p* = 6.00 × 10^−4^). Among sex hormones, we observed positive genetic correlations between SHBG and both estradiol (*p* = 1.00 × 10^−4^) and testosterone (*p* = 1.99 × 10^−85^) at the Bonferroni threshold of significance. When examining the genetic overlap between thyroid hormones and sex hormones, we observed suggestive, nominally significant positive genetic correlations between testosterone and both FT3 (*p* = 3.00 × 10^−3^) and FT4 (*p* = 7.90 × 10^−3^), as well as a Bonferroni-significant positive genetic correlation between FT4 and SHBG (*p* = 2.00 × 10^−4^); we additionally observed a suggestive negative genetic correlation between testosterone and TSH (*p* = 4.50 × 10^−3^).

### 3.3. Genetic Correlations Between Endocrine Hormones and Psychiatric Disorders

**Figure 2.**
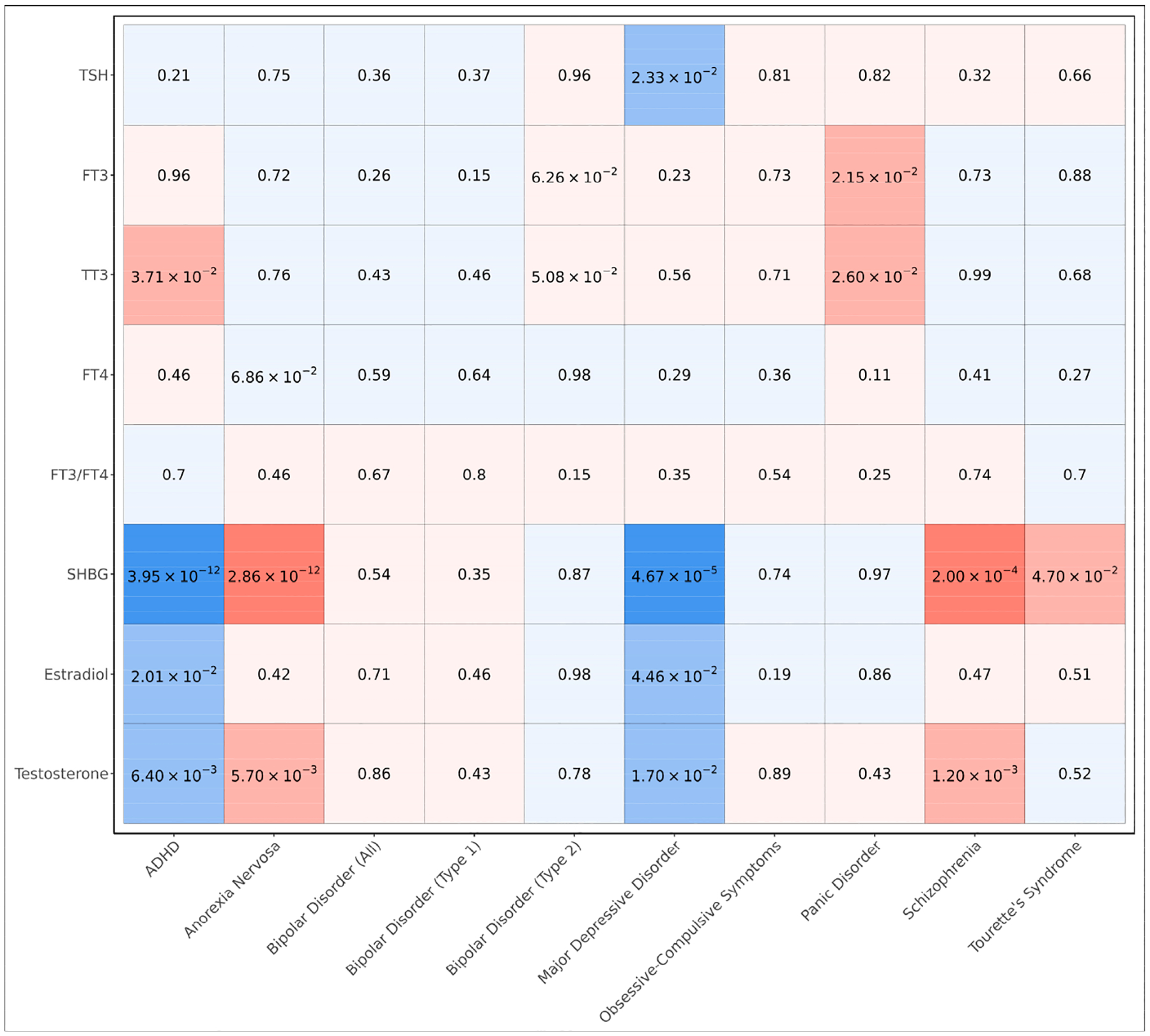
*p*-values of genetic correlations between endocrine hormone metrics and psychiatric disorders. Positive genetic correlations are colored in red and negative genetic correlations are colored in blue. Faint shading indicates a genetic correlation was not statistically significant (*p* ≥ 0.05). Moderate shading indicates a nominally significant genetic correlation (*p* < 0.05). Dark shading indicates a genetic correlation remained significant after adjusting for multiple testing (Bonferroni-adjusted *p* < 0.05 or unadjusted-*p* < 5.56 × 10^−4^).

**Figure 3.**
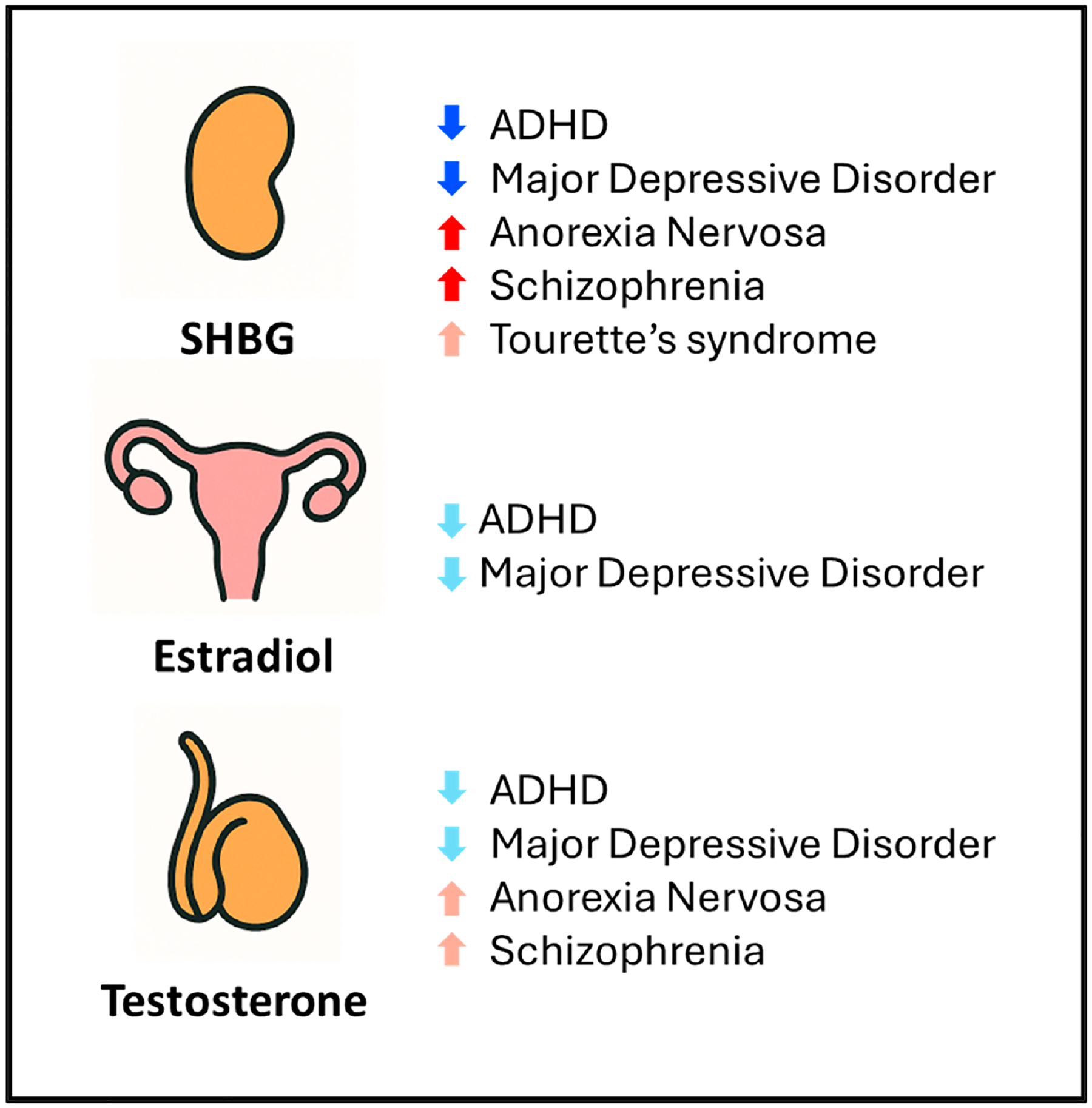
Schematic of genetic correlations between sex hormones and psychiatric disorders. Bonferroni-significant genetic correlations are indicated with upwards-pointing solid red (positive) and downwards-pointing solid blue (negative) arrows. Nominally significant genetic correlations are indicated with upwards-pointing light red (positive) and downwards-pointing light blue (negative) arrows. Abbreviations: SHBG, sex hormone-binding globulin; ADHD, attention-deficit hyperactivity disorder.

To evaluate the shared genetic bases between endocrine hormones and psychiatric disorders, we computed pairwise genetic correlations (Figure 2, Supplementary Table S3). We observed that sex hormones exhibited many genetic correlations with psychiatric disorders including SHBG, which remained statistically significant after utilizing a Bonferroni threshold of significance to account for multiple testing (*p* < 5.56 × 10^−4^); these include negative genetic correlations with ADHD (*p* = 3.95 × 10^−12^) and major depressive disorder (*p* = 4.67 × 10^−5^) and positive genetic correlations with anorexia nervosa (*p* = 2.86 × 10^−12^) and schizophrenia (*p* = 2.00 × 10^−4^) (Figures 2 and 3). Interestingly, we also observed a suggestive, nominally significant positive genetic correlation between SHBG and Tourette’s syndrome (*p* = 4.70 × 10^−2^). In addition, we observed that both testosterone and estradiol had suggestive, nominally significant negative genetic correlations with both ADHD (*p* = 2.01 × 10^−2^ and *p* = 6.40 × 10^−3^, respectively) and major depressive disorder (*p* = 4.46 × 10^−2^ and *p* = 1.70 × 10^−2^, respectively); furthermore, we observed suggestive positive genetic correlations between testosterone and both anorexia nervosa (*p* = 5.70 × 10^−3^) and schizophrenia (*p* = 1.20 × 10^−3^).

Though we did not observe any Bonferroni-significant (*p* < 5.56 × 10^−4^) genetic correlations between thyroid hormone metrics and psychiatric disorders, we observed suggestive positive genetic correlations between TT3 and both ADHD (*p* = 3.71 × 10^−2^) and panic disorder (*p* = 2.60 × 10^−2^) and between FT3 and panic disorder (*p* = 2.15 × 10^−2^) (Figure 2). We additionally observed a suggestive, nominally significant negative genetic correlation between TSH and major depressive disorder (*p* = 2.33 × 10^−2^).

## 4. Discussion

In this study, we systematically examined the shared genetic architecture of common variants underlying (1) different endocrine hormones and (2) the shared etiology between endocrine hormones and psychiatric disorders by computing genetic correlations between endocrine hormone metrics—including thyroid and sex hormones—and ten psychiatric disorders using GWAS summary statistics involving predominantly European-ancestry individuals. After adjusting for multiple testing, we observed extensive genetic correlations among sex hormones and among thyroid hormones separately at a Bonferroni threshold of significance, as well as several genetic correlations between sex and thyroid hormones, suggesting a shared genetic architecture across different endocrine hormones. Furthermore, when examining genetic correlations between endocrine hormones and psychiatric disorders, we identified several genetic correlations at a Bonferroni-threshold of significance, particularly involving sex hormones; notably, SHBG showed statistically significant genetic correlations with multiple psychiatric conditions even after controlling for multiple testing. While the genetic correlations between thyroid hormones and psychiatric disorders did not remain significant after controlling for multiple testing, several suggestive, nominally significant associations were identified, suggesting a potential link in the genetic regulation of thyroid hormone levels and the risk of developing psychiatric disorders.

This study enhances our understanding of the shared genetic basis linking sex hormones and psychiatric disorders by demonstrating that genetic correlations derived from common variants broadly align with prior epidemiological findings. Previous observational and causal inference studies have suggested a potential involvement of SHBG in various psychiatric disorders. Notably, a recent Mendelian randomization study—which utilizes variants as instruments to mimic random assignment—provided evidence that SHBG is causally associated with an increased risk of schizophrenia [30], and males newly diagnosed with a first-episode psychosis have been clinically observed to also exhibit higher SHBG levels than controls [31]. While genetic correlations do not reflect causal relationships and do not control for pleiotropy and potential confounding, we interestingly identified a Bonferroni-significant positive genetic correlation between SHBG levels and schizophrenia, suggesting these have a partially shared genetic basis. Additionally, observational studies have demonstrated that SHBG was negatively correlated with symptoms of ADHD in boys [32] and positively associated with anorexia nervosa amongst kwashiorkor patients [33], which was in general alignment with the negative and positive Bonferroni-significant genetic correlations in our study, respectively. Furthermore, another Mendelian randomization study demonstrated that SHBG had a negative causal association with major depression in males [34], which was supported by our observation of a negative Bonferroni-significant genetic correlation between the two. Interestingly, for each Bonferroni-significant genetic correlation we observed between SHBG and the evaluated psychiatric disorders, either testosterone, estradiol, or both also showed suggestive, nominally significant genetic correlations in the same direction. This pattern observed in our study suggestively indicates a shared genetic architecture among SHBG, testosterone, and estradiol and psychiatric disorders, thereby helping us better understand the relevance of common variants that influence all three of these sex hormones in psychiatric disorders.

Though none of the genetic correlations we computed between thyroid hormone metrics and psychiatric disorders were statistically significant after correcting for multiple testing, several suggestive associations aligned with findings in previous epidemiological studies. We identified a nominal negative genetic correlation between TSH and major depressive disorder, which corroborates a large population-based cross-sectional study in the United States that reported low levels of TSH were associated with a higher odds of clinically relevant depression [35]. In contrast, some of our other observations differed from prior studies; for instance, while it has been reported that a reduction in FT3 levels has been implicated in panic disorders [36,37], we identified a positive genetic correlation between FT3 levels and panic disorder. Additionally, while previous Mendelian randomization studies have indicated that FT4 has a protective effect on bipolar disorders [38–40], including Type 1 bipolar disorder [38], our study did not identify any nominally significant genetic correlations between FT4 and any subtypes of bipolar disorder. These inconsistencies may partially be attributed to the much lower sample sizes utilized in the GWASs for thyroid hormone metrics; this decreased statistical power may potentially result in less reliable genetic correlation estimates. Furthermore, the effects of additional sociodemographic, environmental, and genetic factors beyond common variants in the genome may be insufficiently captured in genetic correlation analyses [41].

In addition, the observed genetic correlations between endocrine hormone metrics and psychiatric disorders suggest that there may be an opportunity to improve the risk prediction of these polygenic disorders via cross-trait modeling. Some recent studies have indicated that multi-level polygenic risk score (PRS) models that incorporate PRS models developed for a host of traits can offer improved predictive accuracy compared to PRS models developed only using data for a single trait or disease [42]. Several endocrine hormone metrics have been evaluated in large-scale, well-powered GWASs and could potentially be leveraged to improve PRS model performance for psychiatric disorders by capturing biological pathways jointly influenced by common variants. Such improvements in the polygenic risk modeling of psychiatric disorders may hold the potential to enable the earlier identification of high-risk individuals, to ultimately inform targeted prevention and screening.

Despite identifying multiple genetic correlations that support a shared genetic architecture between endocrine hormones and psychiatric disorders, this study had several limitations. First, though genetic correlation analyses do provide support for a potentially shared genetic base between two traits, they do not reflect causality as such analyses are susceptible to pleiotropy and confounding. Moreover, for several psychiatric disorders including anorexia nervosa, Tourette’s syndrome, obsessive–compulsive symptoms, and panic disorder, the sample sizes of GWASs we utilized were comparatively low, which may partially explain the general lack of strong genetic correlations of these disorders with endocrine hormones, apart from with SHBG, which derived summary statistics from a well-powered GWAS. Increasing sample sizes involved in future GWASs for these hormone metrics and psychiatric disorders, as well as the inclusion of participants from diverse populations, will be needed to validate the associations identified in this study. Additionally, genetic correlations in this study were restricted to HapMap3 variants, as these are commonly well-imputed across studies and are recommended by the LDSC package for calculating genetic correlations; however, this approach may limit our ability to identify shared genetic architectures located in genomic regions not represented in the HapMap3 panel.

## 5. Conclusions

In conclusion, our study reveals genetic correlations between levels of endocrine hormones, particular sex hormones such as SHBG, and multiple psychiatric disorders, thereby highlighting a shared genetic architecture driven by common variants. These genetic correlations are generally consistent with prior observational and causal inference studies, thus supporting the biological relevance of sex hormones in the etiology of psychiatric disorders. Increasing sample sizes for GWASs of endocrine hormones and psychiatric disorders, the diversity of participants utilized in these studies, as well as the genome coverage to include variants outside of those in the HapMap3 set will help validate the correlations we identified and further improve our understanding of the genetic co-regulation of endocrine hormones and psychiatric disorders.

## Supplementary Material

Supplementary Material

The following supporting information can be downloaded at https://www.mdpi.com/article/10.3390/endocrines6030032/s1, Supplementary Table S1. Characteristics of genome-wide association studies included in this study. Supplementary Table S2. Genetic correlations among different endocrine hormone metrics. Supplementary Table S3. Genetic correlations between endocrine hormone metrics and psychiatric disorders.

## Data Availability

GWAS summary statistics utilized in this study were previously published and are publicly available and for academic use without restriction. GWAS summary statistics for sex hormone metrics utilized in this study are downloadable from the Pan-UK Biobank at https://pan.ukbb.broadinstitute.org/downloads/index.html and were accessed on 18 January 2025. GWAS summary statistics for thyroid function utilized in this study are downloadable from the Thyroidomics Consortium at https://transfer.sysepi.medizin.uni-greifswald.de/thyroidomics/datasets/ and were accessed on 18 January 2025. GWAS summary statistics for psychiatric disorders utilized in this study are downloadable from the Psychiatric Genomics Consortium at https://pgc.unc.edu/for-researchers/download-results/ and were accessed on 18 January 2025. Code for all analyses performed in this study is available at https://github.com/james-li-projects/endocrine_psych_rG and were accessed on 18 January 2025.

## Abbreviations

The following abbreviations are used in this manuscript:

GWAS: Genome-wide association study
ADHD: Attention-deficit hyperactivity disorder
SHBG: Sex hormone-binding globulin
TSH: Thyroid-stimulating hormone
FT3: Free triiodothyronine
TT3: Total triiodothyronine
FT4: Free thyroxine
