# Supplementary material for: Unraveling the Genetic Link Between Endocrine Hormones and Psychiatric Disorders: An Atlas of Genetic Correlations
Source: Endocrines. Author manuscript; Available in PMC 2025 Jul 30. (PMC12290917; doi:10.3390/endocrines6030032)
Supplement: Supplementary Material [file NIHMS2098229-supplement-Supplementary_Material.zip › endocrines-3550899-supplementary.pdf]

**Supplementary Table S1.** Characteristics of genome-wide association studies included in this study.

| Trait Category | Trait | Sample size | PUBMED ID[a] |
|---|---|---|---|
| Endocrine Hormones | Sex Hormone-Binding Globulin | 381,526 individuals | 30305743 (14) |
| | Estradiol | 67,623 individuals | 30305743 (14) |
| | Testosterone | 381,081 individuals | 30305743 (14) |
| | TSH | 271,040 individuals | 38291025 (15) |
| | FT4 | 119,120 individuals | 38291025 (15) |
| | FT3 | 59,061 individuals | 38291025 (15) |
| | TT3 | 15,829 individuals | 38291025 (15) |
| | FT3/FT4 | 51,095 individuals | 38291025 (15) |
| | TT3/FT4 | 15,510 individuals | 38291025 (15) |
| Psychiatric Disorders | Anorexia Nervosa | 16,992 cases / 55,525 controls | 31308545 (16) |
| | Tourette's Syndrome | 4,819 cases / 9,488 controls | 30818990 (17) |
| | Obsessive-Compulsive symptoms | 33,943 individuals | 38548983 (18) |
| | Schizophrenia | 76,755 cases / 243,649 controls | 35396580 (19) |
| | Attention-Deficit Hyperactivity Disorder | 38,691 cases / 186,843 controls | 36702997 (20) |
| | Bipolar Disorder (all) | 41,917 cases / 371,549 controls | 34002096 (21) |
| | Type 1 Bipolar Disorder | 25,060 cases / 371,549 controls | 34002096 (21) |
| | Type 2 Bipolar Disorder | 6,781 cases / 371,549 controls | 34002096 (21) |
| | Major Depressive Disorder | 246,363 cases / 561,190 controls | 30718901 (22) |
| | Panic Disorder | 2,248 cases / 7,992 controls | 31712720 (23) |

Footnote:

[a] citations for each article are placed in parenthesis next to each PUBMED ID

**Supplementary Table S2.** Genetic correlations among different endocrine hormone metrics.

| Hormone #1 | Hormone #2 | Genetic Correlation (rG) | Standard Error of rG | 95% CI of rG | Z-score of rG | P-value of rG |
|---|---|---|---|---|---|---|
| SHBG | FT3 | 0.0648 | 0.0493 | -0.0318, 0.1614 | 1.31 | 0.1889 |
| SHBG | FT4 | 0.1295 | 0.0352 | 0.0605, 0.1985 | 3.68 | 2.00E-04 |
| SHBG | TSH | -0.0424 | 0.0267 | -0.0947, 0.0099 | -1.59 | 0.1123 |
| SHBG | TT3 | 0.0751 | 0.0683 | -0.0588, 0.2090 | 1.1 | 0.2715 |
| SHBG | FT3/FT4 | -0.1031 | 0.0447 | -0.1907, -0.0155 | -2.31 | 0.0211 |
| SHBG | TT3/FT4 | -0.0374 | 0.0564 | -0.1479, 0.0731 | -0.66 | 0.5068 |
| FT3 | TSH | -0.2204 | 0.0643 | -0.3464, -0.0944 | -3.43 | 6.00E-04 |
| FT3/FT4 | FT3 | 0.5218 | 0.0773 | 0.3703, 0.6733 | 6.75 | 1.45E-11 |
| FT3/FT4 | FT4 | -0.759 | 0.0473 | -0.8517, -0.6663 | -16.05 | 4.85E-58 |
| FT3/FT4 | TSH | -0.0136 | 0.076 | -0.1626, 0.1354 | -0.18 | 0.8584 |
| FT3/FT4 | TT3 | 0.3545 | 0.1241 | 0.1113, 0.5977 | 2.86 | 0.0043 |
| Testosterone | SHBG | 0.5933 | 0.0303 | 0.5339, 0.6527 | 19.58 | 1.99E-85 |
| Testosterone | Estradiol | 0.1505 | 0.0779 | -0.0022, 0.3032 | 1.93 | 0.0533 |
| Testosterone | FT3 | 0.1565 | 0.0528 | 0.0530, 0.2600 | 2.96 | 0.003 |
| Testosterone | FT4 | 0.1041 | 0.0392 | 0.0273, 0.1809 | 2.66 | 0.0079 |
| Testosterone | TSH | -0.0845 | 0.0298 | -0.1429, -0.0261 | -2.84 | 0.0045 |
| Testosterone | TT3 | 0.1415 | 0.0787 | -0.0128, 0.2958 | 1.8 | 0.0723 |
| Testosterone | FT3/FT4 | -0.0188 | 0.0471 | -0.1111, 0.0735 | -0.4 | 0.6896 |
| Testosterone | TT3/FT4 | 0.0315 | 0.0644 | -0.0947, 0.1577 | 0.49 | 0.6249 |
| Estradiol | SHBG | 0.2837 | 0.0742 | 0.1383, 0.4291 | 3.82 | 1.00E-04 |
| Estradiol | FT3 | 0.0647 | 0.142 | -0.2136, 0.3430 | 0.46 | 0.6488 |
| Estradiol | FT4 | 0.0356 | 0.1124 | -0.1847, 0.2559 | 0.32 | 0.7517 |
| Estradiol | TSH | -0.0187 | 0.0718 | -0.1594, 0.1220 | -0.26 | 0.7944 |
| Estradiol | TT3 | 0.1945 | 0.2015 | -0.2004, 0.5894 | 0.97 | 0.3346 |
| Estradiol | FT3/FT4 | 0.0539 | 0.1242 | -0.1895, 0.2973 | 0.43 | 0.6644 |
| Estradiol | TT3/FT4 | 0.1073 | 0.2079 | -0.3002, 0.5148 | 0.52 | 0.606 |
| FT4 | FT3 | 0.1401 | 0.1143 | -0.0839, 0.3641 | 1.23 | 0.2203 |
| FT4 | TSH | -0.1211 | 0.0698 | -0.2579, 0.0157 | -1.73 | 0.0829 |
| FT4 | TT3 | 0.0056 | 0.1074 | -0.2049, 0.2161 | 0.05 | 0.9584 |
| TT3 | FT3 | 0.4681 | 0.1458 | 0.1823, 0.7539 | 3.21 | 0.0013 |
| TT3 | TSH | 0.0238 | 0.0859 | -0.1446, 0.1922 | 0.28 | 0.7821 |
| TT3/FT4 | FT3 | 0.1486 | 0.1381 | -0.1221, 0.4193 | 1.08 | 0.2818 |
| TT3/FT4 | FT4 | -0.6675 | 0.0771 | -0.8186, -0.5164 | -8.66 | 4.83E-18 |
| TT3/FT4 | TSH | 0.1135 | 0.0896 | -0.0621, 0.2891 | 1.27 | 0.2056 |
| TT3/FT4 | TT3 | 0.794 | 0.0699 | 0.6570, 0.9310 | 11.36 | 6.91E-30 |
| TT3/FT4 | FT3/FT4 | 0.7105 | 0.1103 | 0.4943, 0.9267 | 6.44 | 1.20E-10 |

**Supplementary Table S3.** Genetic correlations between endocrine hormone metrics and psychiatric disorders.

| Hormone | Psychiatric Disorder | Genetic Correlation (rG) | Standard Error of rG | 95% CI of rG | Z-score of rG | P-value of rG |
|---|---|---|---|---|---|---|
| SHBG | ADHD | -0.1703 | 0.0245 | -0.2183, -0.1223 | -6.95 | 3.95E-12 |
| SHBG | Schizophrenia | 0.0842 | 0.0225 | 0.0401, 0.1283 | 3.74 | 2.00E-04 |
| SHBG | Tourette's Syndrome | 0.0763 | 0.0384 | 0.0010, 0.1516 | 1.99 | 0.047 |
| SHBG | Major Depressive Disorder | -0.0806 | 0.0198 | -0.1194, -0.0418 | -4.07 | 4.67E-05 |
| SHBG | Obsessive-Compulsive Symptoms | -0.0303 | 0.0907 | -0.2081, 0.1475 | -0.33 | 0.7383 |
| SHBG | Bipolar Disorder (Type 1) | 0.0273 | 0.0294 | -0.0303, 0.0849 | 0.93 | 0.3532 |
| SHBG | Bipolar Disorder (Type 2) | -0.0067 | 0.0406 | -0.0863, 0.0729 | -0.17 | 0.8691 |
| SHBG | Bipolar Disorder (All) | 0.0164 | 0.0266 | -0.0357, 0.0685 | 0.62 | 0.5363 |
| SHBG | Panic Disorder | -0.0026 | 0.068 | -0.1359, 0.1307 | -0.04 | 0.9696 |
| SHBG | Anorexia Nervosa | 0.2406 | 0.0344 | 0.1732, 0.3080 | 6.99 | 2.86E-12 |
| FT3 | ADHD | 0.0032 | 0.058 | -0.1105, 0.1169 | 0.06 | 0.9561 |
| FT3 | Schizophrenia | -0.0169 | 0.0482 | -0.1114, 0.0776 | -0.35 | 0.7254 |
| FT3 | Tourette's Syndrome | -0.0131 | 0.0892 | -0.1879, 0.1617 | -0.15 | 0.8831 |
| FT3 | Major Depressive Disorder | 0.0553 | 0.0456 | -0.0341, 0.1447 | 1.21 | 0.2256 |
| FT3 | Obsessive-Compulsive Symptoms | 0.0647 | 0.1907 | -0.3091, 0.4385 | 0.34 | 0.7346 |
| FT3 | Bipolar Disorder (Type 1) | -0.0709 | 0.0496 | -0.1681, 0.0263 | -1.43 | 0.1526 |
| FT3 | Bipolar Disorder (Type 2) | 0.1715 | 0.0921 | -0.0090, 0.3520 | 1.86 | 0.0626 |
| FT3 | Bipolar Disorder (All) | -0.0555 | 0.0489 | -0.1513, 0.0403 | -1.13 | 0.2558 |
| FT3 | Panic Disorder | 0.3609 | 0.157 | 0.0532, 0.6686 | 2.3 | 0.0215 |
| FT3 | Anorexia Nervosa | -0.0269 | 0.0749 | -0.1737, 0.1199 | -0.36 | 0.7192 |
| TSH | ADHD | -0.0375 | 0.0298 | -0.0959, 0.0209 | -1.26 | 0.2086 |
| TSH | Schizophrenia | 0.0301 | 0.0302 | -0.0291, 0.0893 | 1 | 0.3197 |
| TSH | Tourette's Syndrome | 0.0227 | 0.0522 | -0.0796, 0.1250 | 0.43 | 0.664 |
| TSH | Major Depressive Disorder | -0.0526 | 0.0232 | -0.0981, -0.0071 | -2.27 | 0.0233 |
| TSH | Obsessive-Compulsive Symptoms | 0.0265 | 0.1091 | -0.1873, 0.2403 | 0.24 | 0.8081 |
| TSH | Bipolar Disorder (Type 1) | -0.0292 | 0.0329 | -0.0937, 0.0353 | -0.89 | 0.3739 |
| TSH | Bipolar Disorder (Type 2) | 0.0029 | 0.0517 | -0.0984, 0.1042 | 0.06 | 0.9559 |
| TSH | Bipolar Disorder (All) | -0.0288 | 0.0313 | -0.0901, 0.0325 | -0.92 | 0.3584 |
| TSH | Panic Disorder | 0.0181 | 0.0808 | -0.1403, 0.1765 | 0.22 | 0.8223 |
| TSH | Anorexia Nervosa | -0.0144 | 0.0457 | -0.1040, 0.0752 | -0.32 | 0.7525 |
| FT3/FT4 | ADHD | -0.0163 | 0.0428 | -0.1002, 0.0676 | -0.38 | 0.7023 |
| FT3/FT4 | Schizophrenia | 0.0245 | 0.0738 | -0.1201, 0.1691 | 0.33 | 0.7398 |
| FT3/FT4 | Tourette's Syndrome | -0.0303 | 0.0789 | -0.1849, 0.1243 | -0.38 | 0.7007 |
| FT3/FT4 | Major Depressive Disorder | 0.0371 | 0.0394 | -0.0401, 0.1143 | 0.94 | 0.3462 |
| FT3/FT4 | Obsessive-Compulsive Symptoms | 0.1128 | 0.1855 | -0.2508, 0.4764 | 0.61 | 0.543 |
| FT3/FT4 | Bipolar Disorder (Type 1) | 0.0133 | 0.0527 | -0.0900, 0.1166 | 0.25 | 0.801 |
| FT3/FT4 | Bipolar Disorder (Type 2) | 0.1438 | 0.0988 | -0.0498, 0.3374 | 1.46 | 0.1455 |
| FT3/FT4 | Bipolar Disorder (All) | 0.0229 | 0.0545 | -0.0839, 0.1297 | 0.42 | 0.6749 |
| FT3/FT4 | Panic Disorder | 0.1152 | 0.1007 | -0.0822, 0.3126 | 1.14 | 0.2524 |

| | | | | | | |
|---|---|---|---|---|---|---|
| FT3/FT4 | Anorexia Nervosa | 0.0555 | 0.0751 | -0.0917, 0.2027 | 0.74 | 0.46 |
| Testosterone | ADHD | -0.0818 | 0.03 | -0.1406, -0.0230 | -2.73 | 0.0064 |
| Testosterone | Schizophrenia | 0.0957 | 0.0295 | 0.0379, 0.1535 | 3.24 | 0.0012 |
| Testosterone | Tourette's Syndrome | -0.0296 | 0.0457 | -0.1192, 0.0600 | -0.65 | 0.517 |
| Testosterone | Major Depressive Disorder | -0.0532 | 0.0223 | -0.0969, -0.0095 | -2.39 | 0.017 |
| Testosterone | Obsessive-Compulsive Symptoms | 0.0164 | 0.1205 | -0.2198, 0.2526 | 0.14 | 0.8918 |
| Testosterone | Bipolar Disorder (Type 1) | 0.0233 | 0.0296 | -0.0347, 0.0813 | 0.79 | 0.4321 |
| Testosterone | Bipolar Disorder (Type 2) | -0.013 | 0.0474 | -0.1059, 0.0799 | -0.27 | 0.7845 |
| Testosterone | Bipolar Disorder (All) | 0.0048 | 0.0284 | -0.0509, 0.0605 | 0.17 | 0.8644 |
| Testosterone | Panic Disorder | 0.0564 | 0.0718 | -0.0843, 0.1971 | 0.79 | 0.4319 |
| Testosterone | Anorexia Nervosa | 0.1162 | 0.042 | 0.0339, 0.1985 | 2.77 | 0.0057 |
| Estradiol | ADHD | -0.2105 | 0.0905 | -0.3879, -0.0331 | -2.33 | 0.0201 |
| Estradiol | Schizophrenia | 0.0745 | 0.1037 | -0.1288, 0.2778 | 0.72 | 0.4725 |
| Estradiol | Tourette's Syndrome | 0.0902 | 0.1383 | -0.1809, 0.3613 | 0.65 | 0.5142 |
| Estradiol | Major Depressive Disorder | -0.1924 | 0.0958 | -0.3802, -0.0046 | -2.01 | 0.0446 |
| Estradiol | Obsessive-Compulsive Symptoms | -0.3831 | 0.2908 | -0.9531, 0.1869 | -1.32 | 0.1878 |
| Estradiol | Bipolar Disorder (Type 1) | 0.0669 | 0.0907 | -0.1109, 0.2447 | 0.74 | 0.4609 |
| Estradiol | Bipolar Disorder (Type 2) | -0.0046 | 0.1621 | -0.3223, 0.3131 | -0.03 | 0.9775 |
| Estradiol | Bipolar Disorder (All) | 0.0352 | 0.0937 | -0.1485, 0.2189 | 0.38 | 0.7068 |
| Estradiol | Panic Disorder | -0.0347 | 0.2015 | -0.4296, 0.3602 | -0.17 | 0.8632 |
| Estradiol | Anorexia Nervosa | 0.0906 | 0.1125 | -0.1299, 0.3111 | 0.81 | 0.4209 |
| FT4 | ADHD | 0.0226 | 0.0305 | -0.0372, 0.0824 | 0.74 | 0.4584 |
| FT4 | Schizophrenia | -0.041 | 0.0498 | -0.1386, 0.0566 | -0.82 | 0.4101 |
| FT4 | Tourette's Syndrome | -0.0634 | 0.0573 | -0.1757, 0.0489 | -1.11 | 0.2688 |
| FT4 | Major Depressive Disorder | -0.0372 | 0.035 | -0.1058, 0.0314 | -1.06 | 0.2877 |
| FT4 | Obsessive-Compulsive Symptoms | -0.1327 | 0.1451 | -0.4171, 0.1517 | -0.91 | 0.3604 |
| FT4 | Bipolar Disorder (Type 1) | -0.0175 | 0.037 | -0.0900, 0.0550 | -0.47 | 0.6355 |
| FT4 | Bipolar Disorder (Type 2) | -0.0016 | 0.0766 | -0.1517, 0.1485 | -0.02 | 0.9829 |
| FT4 | Bipolar Disorder (All) | -0.0205 | 0.0385 | -0.0960, 0.0550 | -0.53 | 0.5938 |
| FT4 | Panic Disorder | 0.135 | 0.0834 | -0.0285, 0.2985 | 1.62 | 0.1056 |
| FT4 | Anorexia Nervosa | -0.0964 | 0.0529 | -0.2001, 0.0073 | -1.82 | 0.0686 |
| TT3 | ADHD | 0.1748 | 0.0838 | 0.0106, 0.3390 | 2.09 | 0.0371 |
| TT3 | Schizophrenia | -8.00E-04 | 0.0698 | -0.1376, 0.1360 | -0.01 | 0.9908 |
| TT3 | Tourette's Syndrome | -0.0499 | 0.1222 | -0.2894, 0.1896 | -0.41 | 0.6826 |
| TT3 | Major Depressive Disorder | 0.0376 | 0.064 | -0.0878, 0.1630 | 0.59 | 0.5574 |
| TT3 | Obsessive-Compulsive Symptoms | 0.1015 | 0.2692 | -0.4261, 0.6291 | 0.38 | 0.7063 |
| TT3 | Bipolar Disorder (Type 1) | -0.0538 | 0.0736 | -0.1981, 0.0905 | -0.73 | 0.4643 |
| TT3 | Bipolar Disorder (Type 2) | 0.2655 | 0.1359 | -0.0009, 0.5319 | 1.95 | 0.0508 |
| TT3 | Bipolar Disorder (All) | -0.0572 | 0.0725 | -0.1993, 0.0849 | -0.79 | 0.4296 |
| TT3 | Panic Disorder | 0.5051 | 0.2268 | 0.0606, 0.9496 | 2.23 | 0.026 |
| TT3 | Anorexia Nervosa | -0.0303 | 0.1003 | -0.2269, 0.1663 | -0.3 | 0.7624 |
